# Supplementary material for: The chromokinesin Kid (KIF22) forms a homodimer, moves processively along microtubules, and transports double-stranded DNA
Source: eLife. 2026 Sep 16;13:RP102828. doi: 10.7554/eLife.102828 (PMC13581288; doi:10.7554/eLife.102828)
Supplement: Supplementary file 6 [file elife-102828-fig2-data3.zip › Figure 2 Source data 3.pdf]

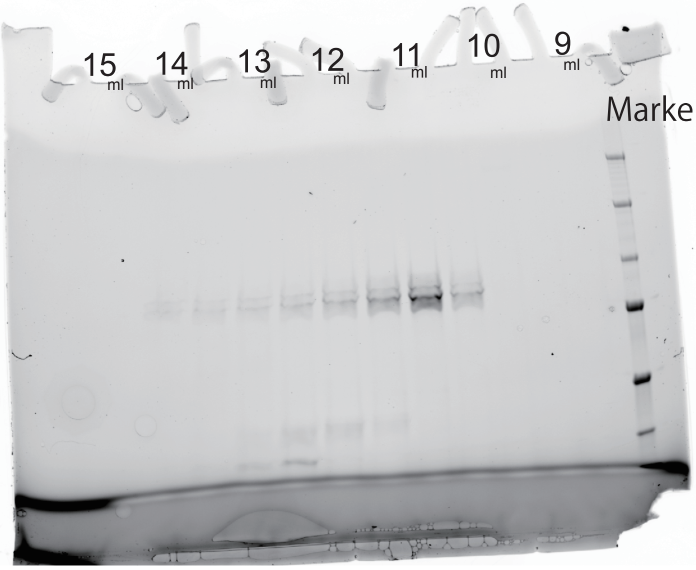
15 ml
14 ml
13 ml
12 ml
11 ml
10 ml
9 ml
Marker
