# Supplementary material for: The chromokinesin Kid (KIF22) forms a homodimer, moves processively along microtubules, and transports double-stranded DNA
Source: eLife. 2026 Sep 16;13:RP102828. doi: 10.7554/eLife.102828 (PMC13581288; doi:10.7554/eLife.102828)
Supplement: Supplementary file 9 [file elife-102828-fig3-data3.zip › Figure3 source_data 3.pdf]

Figure 3B

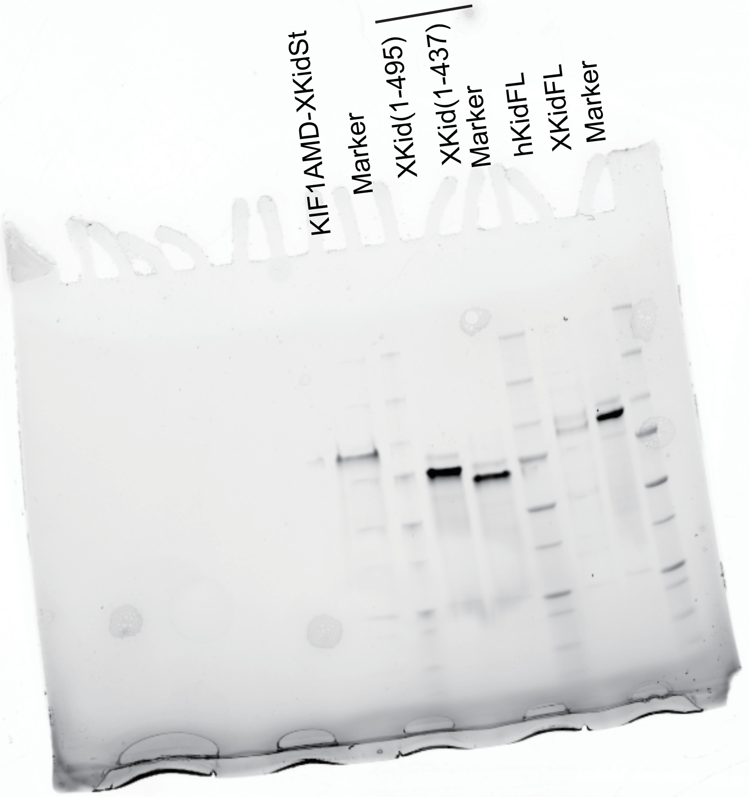

KIF1AMD-XKidSt
Marker
XKid(1-495)
XKid(1-437)
Marker
hKidFL
XKidFL
Marker
