# Supplementary material for: The chromokinesin Kid (KIF22) forms a homodimer, moves processively along microtubules, and transports double-stranded DNA
Source: eLife. 2026 Sep 16;13:RP102828. doi: 10.7554/eLife.102828 (PMC13581288; doi:10.7554/eLife.102828)
Supplement: Peer review history

# Editor report: eLife Assessment

**DOI:** 10.7554/eLife.102828.3.sa0

**Authors:** Kassandra M Ori-McKenney

**Affiliations:** University of California, Davis United States

**Keywords:** Convincing, Important

This **important** study clarifies the mechanism by which the kinesin-10 motor protein, chromosome-associated kinesin, Kid (KIF22), enables chromosome movement during mitosis, demonstrating that human and Xenopus Kid proteins function as processive, homodimeric kinesins capable of processive microtubule plus-end motility. The **convincing** work highlights that Kid can recruit and transport duplex DNA along microtubules via its conserved C-terminal DNA-binding domain, revising our understanding of chromokinesins' role in chromosome motility during mitosis. It will be of interest to those in the molecular motor community working at the molecular, cellular, and organismal levels.

# Referee report: Reviewer #1 (Public review)

**DOI:** 10.7554/eLife.102828.3.sa1

[Editors' note: this version has been assessed by the Reviewing Editor without further input from the original reviewers. The authors have addressed the comments raised in the previous round of review.]

Summary:

Mitotic kinesins carry out crucial roles in intracellular motility and mitotic spindle organization. Although many mitotic kinesins have been extensively studied, a few conserved mitotic motors remain poorly explored, including chromosome-associated kinesins. Here, Furusaki et al reconstitute recombinant chromosome-associated kinesin or chromokinesin (Kid) and reveal processive plus-end motility along microtubules. The authors purify multiple versions of Kid, revealing dimeric organization and their processive microtubule plus-ended motility which depends on their conserved motor domains, neck linkers, and coiled-coil regions. The study reveals for the first time that KID can recruit and transport duplex DNA along microtubules using its conserved C-terminal DNA binding domain. The work provides crucial revised thinking about the mechanisms of Chromokinesins mitosis as physical processive motors that mobilize chromosomes towards the microtubule plus ends in early metaphase.

Strengths:

The authors reconstitute multiple chromosome-associated kinesin (KID) orthologs from Xenopus and humans with microtubules and determine their oligomerization. The study shows how coiled-coil and neck linker regions of KID are essential for its function as its deletion leads to non-processive motility. Chimeras placing the KID coiled-coil and neck linker on the KIF1A motor domain led to the production of a processive recombinant motor supporting the compatibility of their motility mechanisms. The KID c-terminal tail binds and transports only double-stranded DNA and its deletion or single-stranded DNA leads to defects in this activity.

# Referee report: Reviewer #2 (Public review)

**DOI:** 10.7554/eLife.102828.3.sa2

Summary:

Previous work in the field highlighted the role of the kinesin-10 motor protein Kid (KIF22) in the polar ejection force during prometaphase. However, the biochemical and biophysical properties of Kid that enabled it to serve in this role were unclear. The authors demonstrate that human and xenopus Kid proteins are processive kinesins that function as homodimeric molecules. The data are solid and support the findings although the text could use some editing to improve clarity.

Strengths:

A highlight of the work is the reconstitution of DNA transport in vitro.

A second highlight is the demonstration that the monomer vs dimer state is dependent on protein concentration.

# Author comment: Author response

**DOI:** 10.7554/eLife.102828.3.sa3

**Authors:** Shinsuke Niwa, Natsuki Furusaki, Tomoki Kita, Yuki Suzuki, Kyoko Chiba

**Affiliations:** Graduate School of Life Sciences, Tohoku University Sendai Japan; Graduate School of Life Sciences, Tohoku University Sendai Japan; Tohoku University Sendai Japan; Mie University Tsu Japan; Tohoku University Sendai Japan

The following is the authors’ response to the original reviews.

In this revised manuscript, we added new analyses of the DNA-binding tail domain of Kid. AlphaFold 3 predictions suggested that dimeric Kid interacts more stably with double-stranded DNA than monomeric Kid. To experimentally test this prediction, we introduced a point mutation into a critical residue predicted to contribute to DNA binding. Consistent with the AlphaFold 3 model, this mutation abolished the interaction between Kid and DNA.

We also extended our DNA transport assays by testing DNA substrates of different lengths. In addition to 100-bp double-stranded DNA, full-length Kid transported 1,000-bp and 2,000-bp DNA molecules along microtubules in vitro. These findings show that Kid can transport longer duplex DNA substrates than those initially tested, although these substrates do not fully recapitulate the organization of condensed chromatin.

Furthermore, we performed dual-color imaging using independently purified Kid-mScarlet3 and Kid-mStayGold proteins. We consistently observed co-migration of the two fluorescently labeled Kid molecules along microtubules, supporting the conclusion that Kid forms dimers on microtubules.

**Public Reviews:**

**Reviewer #1 (Public review):**

Summary:

Mitotic kinesins carry out crucial roles in intracellular motility and mitotic spindle organization. Although many mitotic kinesins have been extensively studied, a few conserved mitotic motors remain poorly explored, including chromosome-associated kinesins. Here, Furusaki et al reconstitute recombinant chromosome-associated kinesin or chromokinesin (Kid) and reveal processive plus-end motility along microtubules. The authors purify multiple versions of Kid, revealing dimeric organization and their processive microtubule plus-ended motility which depends on their conserved motor domains, neck linkers, and coiled-coil regions. The study reveals for the first time that KID can recruit and transport duplex DNA along microtubules using its conserved C-terminal DNA binding domain. The work provides crucial revised thinking about the mechanisms of Chromokinesins mitosis as physical processive motors that mobilize chromosomes towards the microtubule plus ends in early metaphase.

Strengths:

The authors reconstitute multiple chromosome-associated kinesin (KID) orthologs from Xenopus and humans with microtubules and determine their oligomerization. The study shows how coiled-coil and neck linker regions of KID are essential for its function as its deletion leads to non-processive motility. CHimeras placing the KID coiled-coil and neck linker on the KIF1A motor domain led to the production of a processive recombinant motor supporting the compatibility of their motility mechanisms. The KID c-terminal tail binds and transports only double-stranded DNA and its deletion or single-stranded DNA leads to defects in this activity.

Thank you very much.

Weaknesses:

A minor weakness in the studies is that they do not resolve the mechanisms of KID in binding large duplex DNA molecules or condensed chromatin. The authors suggest a model in which KID forms multimers along large chromosomes that lead to their transport, but this model was not directly tested.

We agree with the reviewer that our study does not directly resolve how Kid binds large duplex DNA molecules or condensed chromatin. In the revised manuscript, we have therefore softened our model and now present the idea that multiple Kid dimers act along chromosomes as a possible mechanism rather than a demonstrated conclusion. To strengthen the mechanistic basis of DNA binding, we added AlphaFold 3-based analysis of the Kid DNA-binding tail domain and experimentally tested a predicted DNA-binding residue. Mutation of this residue abolished Kid–DNA binding, supporting the proposed role of the tail domain in DNA engagement. We also added dual-color imaging experiments showing co-migration of independently purified Kid-mScarlet3 and Kid-mStayGold on microtubules, supporting dimer formation on microtubules. We now explicitly state that future studies using chromatinized DNA or chromosome-like substrates will be required to determine how Kid interacts with condensed chromatin in a cellular context.

**Reviewer #2 (Public review):**

Summary:

Previous work in the field highlighted the role of the kinesin-10 motor protein Kid (KIF22) in the polar ejection force during prometaphase. However, the biochemical and biophysical properties of Kid that enabled it to serve in this role were unclear. The authors demonstrate that human and xenopus Kid proteins are processive kinesins that function as homodimeric molecules. The data are solid and support the findings although the text could use some editing to improve clarity.

Strengths:

A highlight of the work is the reconstitution of DNA transport in vitro.

A second highlight is the demonstration that the monomer vs dimer state is dependent on protein concentration.

Thank you very much.

Weaknesses:

The authors make several assumptions of the monomer vs dimer state of various Kid constructs without verifying the protein state using e.g. size exclusion chromatography and/or nanophotometry.

We newly added mass photometry analysis in Figure 3 and Figure 5.

They also make statements about monomer-to-dimer transitions on the microtubule without showing or quantifying the data.

We performed dual color imaging to show the assembly of Kid monomers on microtubules.

The discussion needs to better put the work into context regarding the ability of non-processive motors to work in teams (formerly thought to be the case for Kid) and how their findings on Kid change this prevailing view in the case of polar ejection force.

We have revised the Discussion to better place our findings in the context of collective motor function and polar ejection force generation. Previous biochemical studies led to the prevailing model that Kid is a monomeric and non-processive chromokinesin. Under this model, sustained chromosome movement would require many Kid monomers distributed along chromosome arms to act collectively. Our findings revise this view. We show that full-length Kid forms homodimers, moves processively along microtubules, and directly transports double-stranded DNA. Thus, the elementary force-generating unit of Kid is unlikely to be a non-processive monomer. Instead, a single Kid dimer may act as a processive DNA-bound motor. In the context of mitotic chromosomes, multiple processive Kid dimers bound along chromosome arms could cooperate to generate chromosome-scale polar ejection forces. We have clarified in the Discussion that our model does not exclude ensemble behavior. Rather, it changes the nature of the proposed ensemble from many non-processive monomers to multiple processive dimers.

The authors also do not mention previous work on kinesins with non-conventional neck linker/neck coil regions that have been shown to move processively. Their work on Kid needs to be put into this context.

We thank the reviewer for this important suggestion. We have revised the Discussion to place Kid in the broader context of processive kinesins with non-conventional neck linker or neck coil regions. We now discuss previous work showing that neck-linker length strongly influences kinesin processivity, and that changes in neck-linker length alter the run length and motility properties of kinesin-1, kinesin-2, and other N-terminal kinesins (Shastry and Hancock, 2010; Shastry and Hancock, 2011).

We also discuss studies showing that longer or non-conventional neck linker regions can provide additional functions beyond supporting processive stepping. For example, kinesin-2 can bypass Tau and other microtubule-bound obstacles by protofilament switching, and the neck linker of the mitotic kinesin KIF18A contributes to obstacle navigation within the mitotic spindle (Hoeprich et al., 2014; Malaby et al., 2019).

In this context, we now emphasize that Kid has an exceptionally long and flexible neck linker, approximately four times longer than that of kinesin-1. Despite this non-canonical architecture, the Kid neck linker and coiled-coil region support processive motility, as shown by the processive movement of the KIF1A–Kid chimera. We therefore propose that Kid represents a non-conventional processive chromokinesin whose extended neck linker may help it move along crowded spindle microtubules while remaining attached to DNA or chromatin. We have also stated that this possibility remains to be tested directly.

**Recommendations for the authors:**

**Reviewer #1 (Recommendations for the authors):**

Furusaki et al reconstitute effectively the chromosome-associated kinesin. The studies are well performed and effectively controlled with few minor suggestions

The studies generally lack a few minor items that would improve the current work:

(1) Alpha fold or coiled-coil predictions of the c-terminal region characterizing its organization or the nature of its interaction site with DNA. These should aid the presentation of the work and help refine the boundaries for coiled coils and the DNA binding domain.

We thank the reviewer for this helpful suggestion. In the revised manuscript, we added AlphaFold 3-based structural predictions and coiled-coil predictions for the C-terminal region of Kid (Figure 7). These analyses helped define the predicted DNA-binding tail domain more clearly. The AlphaFold 3 model also suggested a potential DNA-interaction surface within the C-terminal DNA-binding region. We have incorporated these predictions into the revised figure and modified the text to clarify the domain organization of Kid.

(2) The DNA transport motor activity is quite interesting and extending those studies to cover larger segments of DNA which may bind multiple kid motors would be very interesting.

We thank the reviewer for this helpful suggestion. In the revised manuscript, we extended our DNA transport assays using longer double-stranded DNA fragments. In addition to the 100-bp DNA substrate, we tested 1,000-bp and 2,000-bp DNA fragments. Full-length Kid was able to transport both 1,000-bp and 2,000-bp double-stranded DNA along microtubules in vitro. These new data are now included in Figure 6F–I. Interestingly, the motile parameters of 1,000-bp and 2,000-bp DNA were comparable to those observed with 100-bp DNA. This result suggests that, under our reconstituted assay conditions, increasing DNA length does not substantially enhance the apparent transport velocity or run length. One possible explanation is that the interaction between Kid and naked DNA is relatively weak, and thus only one or a small number of Kid molecules productively engage each DNA molecule during transport. Alternatively, additional Kid molecules bound to longer DNA may not strongly affect the measured motility parameters under these assay conditions.

We have added this point to the revised manuscript and now discuss that, in cells, additional factors such as chromatin proteins or chromosome-associated proteins may enhance the avidity or organization of Kid on chromosomes. Future studies using chromatinized DNA or chromosome-like substrates will be needed to determine how multiple Kid molecules engage large chromatin substrates during chromosome congression.

(3) The final model regarding KID transporting chromosomes is probably oversimplified since there are few experiments with large stretches of DNA or chromatin that were not conducted. I suggest longer segments of DNA be studied or the model be redrawn to scale.

We thank the reviewer for this important comment. We agree that the original model was oversimplified because naked DNA fragments do not fully recapitulate the size, structure, or mechanical properties of condensed chromatin or mitotic chromosomes. To address this concern experimentally, we extended our DNA transport assays to longer double-stranded DNA fragments. In addition to 100-bp DNA, we tested 1,000-bp and 2,000-bp DNA fragments and found that full-length hKid can transport both substrates along microtubules in vitro. These new data are now included in Figure 6F–I.

However, we agree that these DNA substrates are still much simpler than condensed chromatin. We have therefore revised the final model to avoid implying that the transport of naked DNA fully explains chromosome-scale movement. The revised model now emphasizes that Kid dimers can directly couple DNA to microtubule-based motility, and that multiple Kid dimers may cooperate on chromosome arms to generate polar ejection forces. We state "This model is not drawn to scale and does not fully represent the structural complexity of condensed chromatin." in the revised legends.

We also state explicitly in the Discussion that future experiments using chromatinized DNA or reconstituted chromosome-like substrates will be required to determine how Kid engages condensed chromatin and generates chromosome-scale forces.

**Reviewer #2 (Recommendations for the authors):**

Major points:

(1) The authors state that XKid(1-437), which lacks the coiled-coil domain, did not show any processive runs yet Figure 3D does show short events that look like directed movement. They do not appear to be diffusive events as they are uni-directional. The authors need to quantify these results (motility, mean square displacement) as they are essential to their arguments about monomer vs dimer state and processive motility.

We thank the reviewer for pointing this out. We agree that, in the original kymographs acquired at lower temporal resolution, some short XKid(1–437) events could appear as directional movements. To address this concern, we repeated the single-molecule motility assays with improved temporal resolution. In the revised manuscript, the kymographs for XKid(1–437) were generated from data acquired at 100 ms per pixel, instead of 3 s per pixel in the previous version. This higher temporal resolution more clearly shows that XKid(1–437) undergoes short, diffusion-like fluctuations rather than sustained unidirectional processive movement.

We also quantified these trajectories by mean-square displacement analysis. XKid(1–495), which retains the coiled-coil domain, showed superlinear MSD scaling with an α value of approximately 1.6, consistent with persistent, directionally biased movement. In contrast, XKid(1–437), which lacks the coiled-coil domain, showed an α value of approximately 0.8, consistent with hindered or diffusion-like motion rather than sustained processive motility.

We have added these higher-temporal-resolution data and MSD quantification to the revised Figure 3 and revised the text accordingly. We now state that XKid(1–437) lacks sustained processive runs, rather than implying that it shows no movement at all.

The authors speculate that the lack of XKid(1-437) processive runs is due to it being unable to form a homodimer. To confirm that the coiled-coil domain is responsible for dimerization, they fuse the coiled-coil to a fluorescent protein. However, the authors should actually show that XKif(1-437) is a monomer by size exclusion chromatography and/or nanophotometry.

We thank the reviewer for this important suggestion. We agree that directly determining the oligomeric state of XKid(1–437) is essential for interpreting the loss of processive motility. We therefore performed mass photometry to measure the molecular mass of purified XKid(1–437).

The mass photometry analysis showed that XKid(1–437) was predominantly monomeric, with no detectable dimer population under the conditions tested. In contrast, XKid(1–495), which retains the coiled-coil domain, showed a minor dimer population, similar to full-length XKid. These results support the conclusion that deletion of the coiled-coil domain disrupts Kid dimerization.

Together with the motility assays and MSD analysis, these data indicate that the coiled-coil domain is required for homodimer formation and sustained processive motility of Kid. We have added these mass photometry data to the revised Figure 3 and revised the text accordingly.

(2) Likewise, the chimeric protein KIF1AMD-XKidSt shows processive motility (Figure 4), and thus authors conclude that it must be a dimer. This should be verified using size exclusion chromatography and/or nanophotometry.

We agree that the oligomeric state of KIF1AMD–XKidSt should be directly examined. We therefore performed mass photometry analysis of purified KIF1AMD–XKidSt.

Mass photometry showed that KIF1AMD–XKidSt behaved similarly to full-length XKid and XKid(1–495). Under the nanomolar concentrations used for mass photometry, KIF1AMD–XKidSt was predominantly monomeric but retained a detectable dimer population. This behavior is consistent with our analysis of full-length Kid and XKid(1–495), which form weak, concentration-dependent dimers. These results indicate that the XKid stalk region in the chimera can support dimer formation, although the dimer is weak under dilute solution conditions.

(3) Lines 236-239, the authors state "in TIRF-based motility assays, although Kid predominantly dissociates into monomers in solution, its direct interaction with microtubules leads to an increased local concentration of Kid on the microtubule surface. As a result, this would facilitate the formation of Kid dimers on the microtubules, leading to processive motility." This statement implies that monomeric motors diffuse on the microtubule surface until they can associate and begin processive motion. Do the authors see such events diffuse motion and/or association of single monomers on microtubules and a resulting change to processive motion? The kymograph in Figure 1C shows only static and motile events for XKid but hKid does appear to undergo diffusive motion. What is the percent of static vs diffusive vs processive events and how does this change with increased concentrations of XKid and HKid?

We thank the reviewer for this important point. We agree that our original statement was too strong, because we did not directly observe monomeric Kid molecules diffusing on microtubules and then associating to initiate processive movement. We have revised the text to clarify that microtubule-dependent dimerization is a model.

To test this model, we performed dual-color imaging using independently purified hKid–mScarlet3 and hKid–mStayGold. These proteins were mixed at 1 pM each, a concentration at which Kid is expected to be predominantly monomeric in solution. We observed co-migration of the two fluorescently labeled Kid proteins along microtubules, supporting the idea that Kid molecules can associate on microtubules and move together.

However, because of the limited temporal resolution of our two-color TIRF system, we could not directly capture the transition from two monomers to a processive dimer on the microtubule surface. We therefore do not quantify the fraction of static, diffusive, and processive events as a function of concentration in this revised manuscript. Instead, we have softened the relevant statement and explicitly note this limitation in the Discussion.

(4) Lines 171-172 - optimal length of neck linker for coordination of the two motor domains has only been shown for kinesin-1 and kinesin-2. In contrast, there are a number of kinesins that do not have typical neck linker domains yet can achieve processivity. The authors need to discuss this work and put their results with Kid into this context.

As described above, we have revised the Discussion to place Kid in the broader context of processive kinesins with non-conventional neck linker or neck coil regions. We now discuss previous work showing that neck-linker length strongly influences kinesin processivity, and that changes in neck-linker length alter the run length and motility properties of kinesins (Shastry and Hancock, 2010; Shastry and Hancock, 2011).

We also discuss studies showing that longer or non-conventional neck linker regions, such as those of kinesin-2 and KIF18, can provide additional functions beyond supporting processive stepping (Hoeprich et al., 2014; Malaby et al., 2019). In this context, we now emphasize that Kid has an exceptionally long and flexible neck linker, approximately four times longer than that of kinesin-1. We described a possibility that the extended neck linker of Kid may help it move along crowded spindle microtubules while remaining attached to DNA or chromatin while this possibility remains to be tested directly.

Minor points:

(5) Lines 68-69 should note that non-processive motors have been shown to move cargo if they are present in multiple copies of the cargo. This should also be discussed in the Discussion.

We described it in the revised manuscript:

“Under this model, sustained chromosome movement would require many Kid monomers distributed along chromosome arms to act collectively.”

“This model preserves the likely importance of motor ensembles on large chromatin, but changes the nature of the ensemble from many non-processive monomers to multiple processive dimers.”

(6) For Figure 4, does the KIF1AMD-XKidSt chimeric protein contain both the stalk (coiled-coil?) and tail (DNA binding?) regions of XKid or just the stalk as shown in the schematic?

We included coiled-coil domain only.

(7) For Figure 5, please provide a schematic for XKid(delta tail).

We now added Alphafold 3 data.

**Senior Editor:**

Along the lines of reviewer #2's request to put the results in the context of existing knowledge, please consider whether you want to cite Pike et al. 2018 (https://doi.org/10.1126/scisignal.aaq1060; some evidence for dimerization in Fig. 4) and Walker et al. 2019 (https://pubs.acs.org/doi/10.1021/acs.biochem.9b00011).

We have cited these papers in the revised manuscript. These are consistent with our finding that Kid can form dimer at higher concentration while dissociate to monomers in lower concentrations.
